# Development of Inhalable Bacteriophage Liposomes Against *Pseudomonas aeruginosa*

**DOI:** 10.3390/pharmaceutics17040405

**Published:** 2025-03-24

**Authors:** Shruti S. Sawant, Maizbha Uddin Ahmed, Nathan-Gautham Gantala, Caitlin Chiu, Li Qu, Qi Zhou

**Affiliations:** Department of Industrial and Molecular Pharmaceutics, College of Pharmacy, Purdue University, West Lafayette, IN 47907, USA; sawant2@purdue.edu (S.S.S.); ahmed156@purdue.edu (M.U.A.); ngantala@utexas.edu (N.-G.G.); cgchiu@purdue.edu (C.C.)

**Keywords:** phages, bacteria, liposomes, nebulization, pulmonary delivery, inhalation

## Abstract

**Background:** *Pseudomonas aeruginosa* is one of the major pathogens that cause respiratory infections. The rise of antimicrobial resistance has prompted a need for alternatives to conventional antibiotics. Bacteriophages (phages), natural predators of bacteria, are gaining interest as an alternative therapeutic option against drug-resistant infections. However, phage viability can be lost during manufacturing and delivery. Recent studies show that phages can be taken up by lung epithelial cells, which makes fewer phages available for antibacterial action against extracellular bacteria *P. aeruginosa* in the airways. **Methods:** In this study, we encapsulated phages in liposomes using thin film hydration. The effect of processing conditions and phage loading titer on the phage encapsulation and viability was studied. The impact of nebulization on phage viability was tested using an air-jet nebulizer (PARI-LC Plus). Phage cellular uptake was evaluated using an in vitro H441 lung epithelial cell model, grown at the air–liquid interface. **Results:** Our results demonstrate favorable encapsulation (58 ± 6.02%) can be achieved with minimum loss in phage titer (0.64 ± 0.21 log) by using a low phage titer for hydration. The liposomal formulations exhibited controlled release of phages over 10 h. The formulation also reduced the loss of phage viability during nebulization from 1.55 ± 0.04 log (for phage suspension) to 1.08 ± 0.05 log (for phage liposomes). Encapsulation of phages in liposomes enabled a two-fold reduction in phage cellular uptake and longer extracellular phage retention in the human lung epithelial cell monolayer. **Conclusions:** Our results indicate that liposomal encapsulation favors phage protection and improves phage availability for antibacterial activity. These findings highlight the potential of liposomes for inhaled phage delivery.

## 1. Introduction

The World Health Organization (WHO) has identified antimicrobial resistance (AMR) as one of the top two threats to public health worldwide. In 2019, around 4.95 million deaths were caused due to antimicrobial-resistant bacteria, and more than 1.5 million deaths were linked to lower respiratory tract infections [1]. More than 10 million annual deaths are projected by 2050 [2]. The overuse and misuse of antibiotics have rendered bacteria insensitive to treatment. Ultimately, infections persist, and limited antibiotic options are available to cure the disease. Bacteria prone to rapid development of resistance fall under the category of “ESKAPE” pathogens, which includes *Enterococcus faecium*, *Staphylococcus aureus*, *Klebsiella pneumoniae*, *Acinetobacter baumannii*, *Pseudomonas aeruginosa*, and *Enterobacter* species [3].

Among the ESKAPE pathogens, *Pseudomonas aeruginosa* is a major pathogen that causes lung infections. Resistance to antibiotics in these infections is often linked to high mortality [4,5]. This alarming rise in antibiotic resistance has prompted the need for new antibiotics or alternative strategies against multidrug-resistant bacteria. While antibiotic resistance continues to escalate, recent decades have witnessed a dearth of newer antibiotics. Meanwhile, bacteriophage (or phage) therapy is gaining attention as an alternative to conventional antibiotics. Phages are naturally occurring viruses that can infect and kill their specific bacterial host [6]. The United States Food and Drug Administration (FDA) considers phage as a biological product; however, there is no approved product in the United States yet. There are several ongoing clinical trials for phage therapies, with four out of five trials against *Pseudomonas aeruginosa* respiratory infections focused on inhaled phage therapy [7].

Inhaled phage therapy allows direct delivery of phages into the lumen of the lungs and can kill the bacteria present in the lung airways. Multiple studies have demonstrated the prospective use of phages for lung infections [8,9,10,11,12,13]. The efficacy of phage therapy is highly dependent on the phages available at the site of infection. However, phages may lose their viability when exposed to mechanical stresses during manufacturing, storage, and delivery [14]. Additionally, once delivered into the lungs, phage distribution can be spatially heterogeneous relative to the site of bacterial infection [15]. Recent findings indicate that phages can be internalized by mammalian cells (such as the lung epithelial cells) and represent a “sink” in phage therapy [16]. The lung airways are primarily lined by lung epithelial cells; phage uptake by these epithelial cells can reduce the phages available for killing the extracellular bacteria (e.g., *P. aeruginosa*). To overcome these challenges, advanced formulations are required to protect phages during manufacturing and delivery.

Phage encapsulation in liposomes has been studied for oral therapy. In these studies, liposome encapsulation protects phages from environmental degradation, such as pH and enzymes [17]. Phage liposomes have been used for targeting intracellular infections like *Klebsiella pneumoniae* [18], *Staphylococcus aureus* infections [19], and *Mycobacterium tuberculosis* [20]. Only a single study highlights the use of phage–liposome nanocomplexes for inhaled phage therapy against intracellular *Staphylococcus aureus* infections [19]. The use of liposomes for inhaled phage delivery and for targeting extracellular infections remains unexplored. The present study focuses on extracellular bacteria *P. aeruginosa*, which is found in chronic lung infections (like cystic fibrosis). The objective of this study is to develop inhalable phage liposomes to target extracellular *P. aeruginosa*. We hypothesize that encapsulation of phages in liposomes can offer protection during manufacturing and delivery while facilitating extracellular phage retention. To achieve this, thi study aims to investigate: (i) factors influencing phage encapsulation in liposomes, (ii) the effect on phage aerosolization, and (iii) phage cellular uptake in a lung epithelial cell model.

## 2. Materials and Methods

### 2.1. Materials

Lipids, including hydrogenated soybean phosphatidylcholine (HSPC), 1,2-Distearoyl-sn-glycero-3-phosphoglycerol, sodium salt (DSPG-Na), and *N*-(methylpolyyoxyethylene oxycarbonyl)-1,2-distearoyl-sn-glycero-3-phosphoethanolamine (DSPE-PEG) were procured from NOF America Corporation (White Plains, NY, USA). Labeled lipid 1,2-dipalmitoyl-sn-glycero-3-phoaphoethanolamine-*N*-(lissamine rhodamine B sulfonyl) (ammonium salt) (i.e., 16:0 Liss Rhod PE) was obtained from Avanti Polar Lipids, Inc. (Alabaster, AL, USA). Cholesterol, mannitol, and sodium deoxycholate (SDC) were purchased from Sigma-Aldrich (St. Louis, MO, USA). Sucrose and methanol were purchased from Fisher Scientific (Waltham, MA, USA). Simulated lung fluid with DPPC (Dipalmitoylphosphatidylcholine) was received from Pickering Labs (Mountain View, CA, USA).

Bacteria *Pseudomonas aeruginosa* 95 (ATCC 14211) and its complimentary phage *Pseudomonas aeruginosa* bacteriophage 95 (ATCC 14211-B1) were received from American Type Culture Collection (Manassas, VA, USA) in the form of a freeze-dried powder. Brain Heart Infusion (BHI) media used for bacterial culture and phage propagation was obtained from Becton Dickinson (Franklin Lakes, NJ, USA). Bacteriological agar, radioimmunoprecipitation assay (RIPA) buffer, SYBR Gold nucleic acid gel stain, Hoechst 33342, and Bicinchoninic acid (BCA) protein assay kit were purchased from ThermoFisher Scientific (Rockford, IL, USA).

Cell culture supplies, including Roswell Park Memorial Institute 1640 Medium (RPMI 1640 Medium), Fetal Bovine Serum (FBS), Penicillin (10,000 units/mL)—Streptomycin (10,000 µg/mL) (PenStrep), Insulin-Transferrin-Selenium (ITS, 100×), and Hanks’ Balanced Salt Solution (HBSS) with calcium and magnesium (1×) were purchased from Gibco (Life Technologies Corporation, Eugene, OR, USA). Cell culture flasks and transwell plates (6.5 mm insert, 0.4 µm polyester membrane) were supplied by Corning Incorporated (Kennebunk, ME, USA).

### 2.2. Phage Propagation and Determination of Phage Titer Using Plaque Assay

*Pseudomonas aeruginosa* bacteriophage 95 (ATCC 14211-B1) was propagated in the presence of its complementary host, *Pseudomonas aeruginosa* 95 (ATCC 14211). Phage propagation and determination of phage titer were performed using the double-layer agar assay or plaque assay [21]. Briefly, an isolated bacterial colony was inoculated in BHI media (100 mL), followed by overnight incubation. An aliquot of the overnight culture (2.5% *v*/*v*) was mixed with melted soft agar (0.5% *w*/*v* agar in BHI media) at 45–48 °C. The mixture was poured onto the surface of hard agar (1.5% agar *w*/*v*) in a Petri plate. For propagation, the phage stock was mixed with bacteria and soft agar before pouring into a Petri plate containing hard agar. The plates were incubated overnight at 37 °C to allow plaque formation. Phages were collected by scraping the soft agar layer and mixing it with BHI media. Phage lysates were obtained by separating the bacteria and agar debris by centrifugation, followed by syringe filtration (0.22 µm) [22]. The phage stocks were stored at 4 °C until further use.

The phage titer of phage samples was determined using the same method. An aliquot of overnight bacterial culture (2.5% *v*/*v*) was mixed with molten soft agar and poured on a Petri plate containing hard agar, as discussed above. Serial dilutions of phage samples were prepared, and 10 µL of the dilution was spotted on the soft agar. Plates were incubated at 37 °C for 16–18 h to allow plaque formation. Dilutions giving countable plaques (3–30 plaques) were used to calculate phage titer. Plaque forming units (PFU) multiplied by the dilution factor corresponded to the phage titer, which indicates the number of viable phages present in a sample. The limit of detection for phages was 100 PFU/mL.

### 2.3. Phage Encapsulation in Liposomes

Phage liposomes were prepared using the thin-film hydration method [23,24]. A thin lipid film was prepared by dissolving HSPC, DSPG-Na, DSPE-PEG, and cholesterol (mass ratio 3:2:0.5:1.7) in chloroform (5 mL) [23,24]. The organic solvent was subjected to rotary evaporation at 55 °C using Buchi Rotavapor 300 (Buchi, New Castle, DE, USA) to form a thin lipid film. The lipid film was hydrated with 250 µL phage stock in 3.75 mL of sugar solution (containing 80 mg/mL mannitol and 20 mg/mL sucrose) at 40 °C for one hour. Hydration at two phage loading titers was tested—1.98 × 10^7^ and 5.49 × 10^8^ PFU/mL. Liposomal vesicles formed on hydration were further downsized using syringe filtration (0.44 µm and 0.22 µm), followed by extrusion. Phage liposomes were extruded through a 0.1 µm polycarbonate membrane at 40 °C using a Mini-Extruder (Avanti Polar Lipids, Alabaster, AL, USA).

### 2.4. Phage Encapsulation Efficiency

The liposomes with encapsulated phages were separated from free phages (*C_free_*) by size exclusion chromatography (SEC) using a Sephadex G-25 column [25]. The titer of the encapsulated phages (*C_encapsulated_*) was determined by lysing the liposomes with a mild detergent, 0.01% *v*/*v* sodium deoxycholate (SDC), followed by centrifugation [26]. The phage titer of the free and encapsulated phages was determined using the plaque assay as described previously (Section 2.2). The percentage of phage encapsulation was calculated using the following formula:

$$\%\;Phage\;encapsulation=\left(1-\frac{C_{free}}{C_{encapsulated}+C_{free}}\right)\times 100$$

### 2.5. Particle Characterization of Phage Liposomes

The particle properties of phage liposomes, including the mean particle size, polydispersity index, and zeta potential, were determined using a Malvern Zetasizer Nano ZS90 (Malvern Panalytical, Worcestershire, UK).

### 2.6. Release Studies

The release of encapsulated phages from liposomes was tested in commercially available simulated lung fluid (SLF) containing lung surfactant DPPC (Pickering Labs, Mountain View, CA, USA) to mimic the lung physiological conditions. Briefly, phage liposomes (1 mL, titer 1.35 × 10^6^ PFU/mL) were incubated with SLF (9 mL) in a scintillation vial kept on an orbital shaker at 37 °C. Every 2 h, 1 mL aliquot was collected from the release samples and replenished with an equal volume of SLF (i.e., 1 mL). Each release sample was further processed to determine free phages released, and the total phages present in the release media at every time point. Free phages were separated by subjecting the release samples (0.5 mL) to centrifugation (14,000× *g*, 15 min). Liposomes settled as a pellet and free phages were present in the supernatant. The total phages present in the release media were obtained by lysing the liposomes using SDC (0.01% *v*/*v*). Both free and total phages were quantified using the plaque assay described above (Section 2.2). The percentage of phages released from liposomes at a given time point was determined using the following formula:

$$\%\;Phage\;release\;at\;\mathrm{time}\;‘t’=\frac{Free\;phages\;at\;‘t’}{Total\;phages\;at\;‘t’}\times 100$$

### 2.7. Nebulization Studies

#### 2.7.1. Phage Viability upon Nebulization

The stability of phages on nebulization was tested using a jet nebulizer, the PARI Proneb Max Aerosol system with an LC plus nebulizer (Pari GmbH, Starnberg, Germany). The impact of nebulization on phage viability was studied by nebulizing 1 mL of phage suspension/liposomes (phage titer ranging from 0.8 to 1.8 × 10^6^ PFU/mL) for 5 min. The nebulized droplets were collected using a PARI filter/valve set [27]. Phages deposited on the filter pads were recovered by overnight incubation in 20 mL BHI media at 4 °C. The loss in phage viability was measured using the plaque assay (Section 2.2).

#### 2.7.2. In Vitro Aerosolization Studies

The in vitro aerosolization profile of phage liposomes was assessed using a Multi-Stage Liquid Impinger (MSLI) (Copley, Nottingham, UK). The LC Plus nebulizer cup with the compressor was connected to the MSLI via the induction port. The HCP5 vacuum pump (Copley Scientific, UK) and a DFM 2000 flow meter (Copley Scientific, UK) were used to set the flow rate at 28 L/minute. The cut-off diameter at the given flow rate at Stages 1–4 corresponded to 18.93, 9.90, 4.51, and 2.48 µm, respectively [19]. Each stage of the MSLI consisted of 20 mL water and was allowed to wet all surfaces of the MSLI. Phage suspension/liposomes (1 mL) were nebulized from the PARI LC Plus cup (Pari GmbH, Starnberg, Germany) through the induction port and MSLI stages for a run time of 5 min. After completion of the run, samples from the reservoir cup and stages were collected, followed by the determination of phage titer (as described in Section 2.2). The fine particle fraction (FPF) was measured as the percentage of the phages deposited in Stage 4 and the filter relative to the emitted phages.

### 2.8. In Vitro Phage Cellular Uptake Studies

The H441 cell line is a common cell line representing the distal human lung epithelial cells and was used as a model for in vitro studies [28]. NCI-H441 (American Type Cell Culture—ATCC) was cultured in 75 cm^2^ flasks in cell culture media RPMI-1640 medium supplemented with 10% (*v*/*v*) fetal bovine serum (FBS) and 1% (*v*/*v*) penicillin–streptomycin. Cells were incubated at 37 °C under 5% CO_2_ and routinely passaged on reaching 80% confluency.

Phage cellular uptake and transport were studied using an H441 lung epithelial cell monolayer. Briefly, NCI-H441 cells were grown in a 24-well Corning transwell plate (6.5 mm insert, 0.4 µm polyester membrane, area 0.33 cm^2^). Cells were seeded in the apical chamber of each transwell at a density of 500,000 cells/cm^2^, followed by incubation in media for 24 h at 37 °C. After attachment, cells were polarized with cell culture media supplemented with insulin–transferrin selenium (1% *v*/*v*) and dexamethasone (200 nM) [29]. The cells were incubated with polarization medium in both chambers (apical and basal) for 48 h. On completion of polarization, cells were grown at the air–liquid interface (ALI) for two weeks, with cell growth media (600 µL) only in the basal chamber until a confluent monolayer was formed.

A confluent cell monolayer was formed in two weeks. The transepithelial electrical resistance (TEER) across the cell monolayer was measured using an EVOM voltohmmeter before beginning the experiment. An aliquot of 0.2 mL of phage liposomes or a phage suspension control (2.82 × 10^6^ PFU/mL) was placed in the apical chamber, with HBSS in the basal chamber (0.6 mL). The phages were incubated for four hours, as phage uptake can be observed in a minimum of two hours based on previous studies [16]. At the end of the experiment, samples were collected from both chambers (apical and basal), and cells were washed followed by lysis using radioimmunoprecipitation assay (RIPA) buffer. All the samples were tested for phage titer using plaque assay (Section 2.2), and the protein content of the cell lysates was measured using the bicinchoninic acid (BCA) assay (as per the manufacturer’s protocol).

### 2.9. Phage Distribution Imaging Using Confocal Laser Scanning Microscopy

For confocal imaging, the liposomes were labeled by 1% *w*/*w* labeled lipid lissamine rhodamine B sulfonyl to the lipid composition mentioned earlier. Phages labeled with SYBR Gold were used for hydrating the lipid film. Phages were fluorescently labeled with SYBR Gold stain. Briefly, an aliquot of phage stock (1 mL) was incubated with SYBR-Gold (1× final concentration) at 4 °C. After overnight incubation, excess SYBR-Gold was removed using Amicon Ultracentrifugal filters (Molecular weight cut-off 100 kDa, 4 mL). Multiple centrifugation runs were performed to ensure the complete removal of excess stain [22].

For imaging studies, the H441 cell monolayer was treated with SYBR-Gold labeled phages or labeled liposomes containing encapsulated SYBR-Gold phages. After a 4-h incubation, treatments were removed, and cells were washed. For nucleus staining, cells were stained with Hoechst 33342. The cell monolayer was cut, placed on a glass slide, and covered with an antifade mounting reagent and coverslip. The cell monolayer was imaged using a Nikon-A1Rsi confocal microscope (Nikon America Inc., Melville, NY, USA) with a 60× oil immersion lens. The 3D image was generated by using the Z-stack function to analyse the phage distribution across the entire cell monolayer. Captured images were viewed using Nikon NIS Elements software (version 5.21.00) [30].

### 2.10. Statistical Analysis

All results in this study are expressed as mean ± standard deviation (SD), with three replicates unless otherwise specified. Statistical analysis was performed using the GraphPad Prism software (version 10, GraphPad, San Diego, CA, USA). Statistical tests, including the Student’s *t*-test (paired and unpaired) and Tukey’s multiple comparison test, were used. Statistical differences are indicated by *p* values with their level of significance, (** *p* < 0.01, *** *p* < 0.001, **** *p* < 0.0001, *ns*—no significant difference).

## 3. Results

### 3.1. Development of Phage Liposomes—Effect of Process on Phage Viability

**Figure 1:**
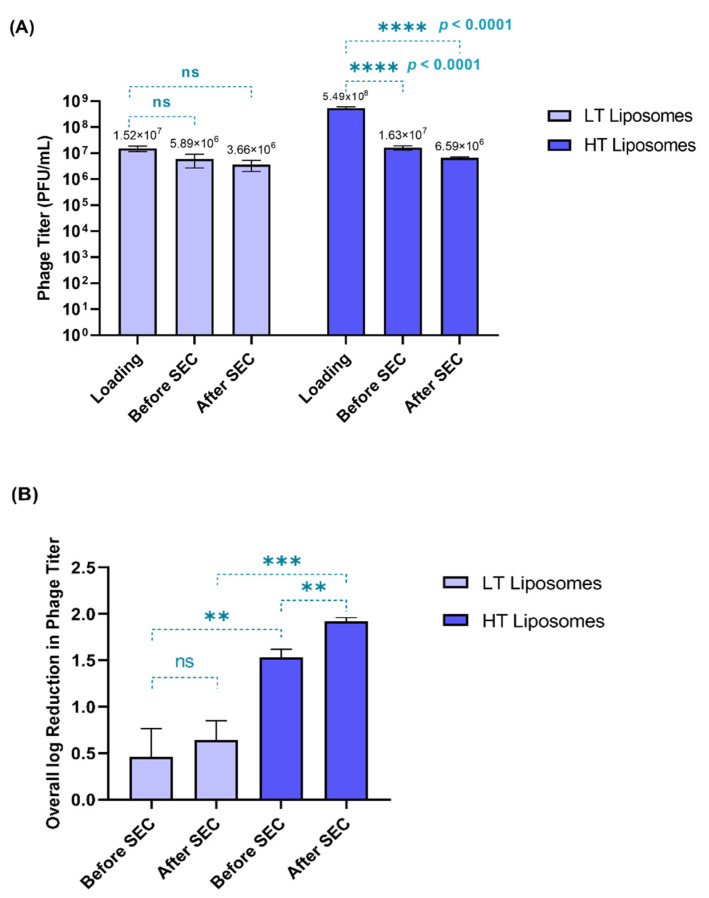
(**A**) Phage viability (in terms of phage titer) measured at different stages in the preparation of liposomes. The phage titer at each stage was compared to the initial loading titer (Tukey’s multiple comparison test, **** *p* < 0.0001, *ns* = no significant difference). (**B**) Overall log reduction in phage titer observed before and after SEC (paired *t*-test, ** *p* < 0.01) among the formulations (unpaired *t*-test) (** *p* < 0.01, *** *p* < 0.001, *ns* = no significant difference). Each data point represents mean ± SD, (*n* = 3).

Two phage loading titers were tested during hydration—1.98 × 10^7^ and 5.49 × 10^8^ PFU/mL—yielding low-titer (LT) and high-titer (HT) liposomes, respectively. Phages can lose their viability due to processing conditions such as hydration temperature and shear stress on extrusion [31]. Therefore, phage viability was tested at different stages in the preparation of liposomes (Figure 1A)—before the separation of free phages (before SEC) and after the separation of free phages (after SEC). Figure 1B compares the overall log reduction during the preparation of LT and HT liposomes.

When compared to the initial loading titer, the HT liposomes showed significantly greater loss (*p* < 0.0001) in phage titer. For the HT liposomes, only 6.59 × 10^6^ PFU/mL was encapsulated, despite using a high loading titer (5.49 × 10^8^ PFU/mL). In contrast, the LT liposomes did not show any significant loss in titer when compared to the initial loading titer. This suggests that most of the phages used in hydration (1.98 × 10^7^ PFU/mL) were encapsulated in the LT liposomes (3.66 × 10^6^ PFU/mL). Figure 1B demonstrates a significant loss in phage titer (*p* < 0.05) in the HT liposomes during SEC, which corresponded to the free phages, whereas no significant loss was observed in the case of the LT liposomes (Figure 1B). This also helps us understand that the presence of free phages in the HT liposomes increases their susceptibility to inactivation due to temperature and shear stresses upon hydration and extrusion. Hence, the overall reduction in phage titer was higher for the HT liposomes (1.92 ± 0.04 log) than the LT liposomes (0.64 ± 0.21 log). Despite the loss in phage viability, it was observed that the encapsulated phage titer was similar in both HT (6.59 × 10^6^ PFU/mL) and LT (3.66 × 10^6^ PFU/mL) liposomes. Conclusively, this study exhibits that low-loading titer used for liposomes can be favorable in minimizing the loss in phage viability.

### 3.2. Particle Characterization and Phage Encapsulation

Phage-encapsulated liposomes have similar particle size and zeta potential, irrespective of the titer used for hydration (Table 1). The particle size distribution was unimodal, suggesting no formation of phage aggregates. The phage encapsulation efficiency was determined at the end of the processing steps by measuring the remaining viable phages. Free phages in the HT liposomes (9.65 × 10^6^ PFU/mL) were four times that of the LT liposomes (2.23 × 10^6^ PFU/mL). However, the encapsulated phage titers were similar in both formulations, regardless of the different loading titers. Therefore, the phage encapsulation was higher in the LT liposomes (58.33 ± 6.02%) compared to the HT liposomes (25.15 ± 5.67%). Due to high encapsulation efficiency and minimum loss in phage viability, the LT liposomes were used in further studies.

### 3.3. Release Studies

**Figure 2:**
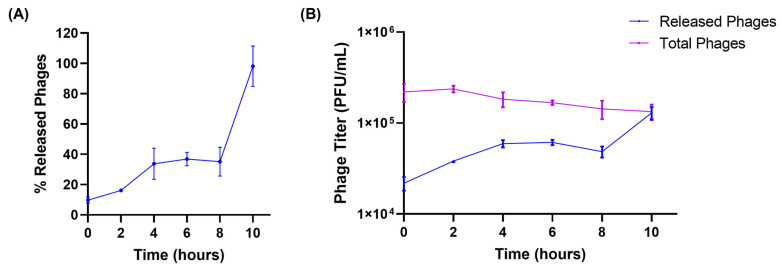
(**A**) Release patterns of phages from liposomes in simulated lung fluid at 37 °C. (**B**) Phage titer of released phages and total viable (encapsulated + released) phages present at different time points (hours) in the release study. Each data point represents mean ± SD, (*n* = 3).

The release of encapsulated phages from liposomes was tested in the simulated lung fluid to better mimic the physiological conditions. Figure 2A illustrates the release of phages from liposomes. At the beginning of the release studies (t = 0 h), a burst release of phages from liposomes, accounting for 10% of phages, was observed. This may be due to the loosely bound phages present on the surface of the liposomes. This was followed by a slow and more controlled release of phages from liposomes. About 35.15 ± 9.50% of the encapsulated phages were released over 8 h. The final phase of phage release was observed from 8 to 10 h, during which all the encapsulated phages (98.13 ± 13.29%) were released into the SLF. The total viable phages (sum of released and encapsulated phages) were measured at every time point, to ensure phages remained stable in the SLF throughout the study. No significant reduction in phage viability was observed while performing release studies in the SLF (Figure 2B). Furthermore, at the end of 10 h, the titer of the released phages was equal to the total viable phages, proving the complete release of encapsulated phages from liposomes.

### 3.4. Nebulization Studies and In Vitro Lung Deposition

**Figure 3:**
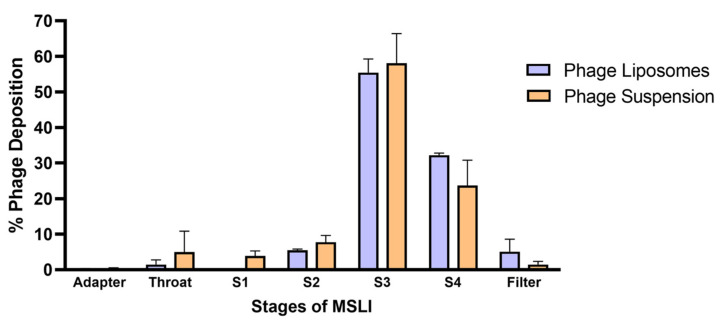
Phage deposition profiles across different stages of the MSLI following nebulization with PARI LC Plus nebulizer. Percentage deposition is relative to the phages recovered after aerosolization through the nebulizer. Each data point represents mean ± SD, (*n* = 3).

An important aspect when developing inhalable phage liposomes was to understand the effect of nebulization on (1) phage viability and (2) deposition of phages in the lungs. The effect on phage viability was assessed by comparing the phage titer before aerosolization and the phages recovered after nebulization. Table 2 summarizes the effect of nebulization on the phage titer of the phage suspension/liposomes. It was observed that the phage suspensions showed a significantly (*** *p* < 0.001) higher loss in phage titer (1.55 ± 0.04 log loss) upon nebulization compared to the liposomes (1.08 ± 0.05 log loss). The in vitro phage deposition was studied using MSLI, and the deposition profile (Figure 3) was obtained by determining the percentage of aerosolized phages deposited at different stages. The fine particle fraction (FPF) refers to the phage aerosols with an aerodynamic diameter of less than 5 µm. FPF accounts for the percentage of phages deposited at stage 4 and filter. Therefore, the FPF of the phage liposomes was 37.32 ± 2.94%, while that of the phage suspension was 25.09 ± 8.01%. No significant differences were observed in FPF between phage suspension and liposomes.

### 3.5. Phage Cellular Uptake—Quantitative Analysis

**Figure 4:**
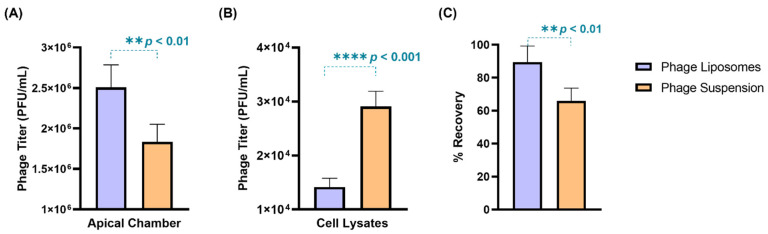
The distribution of phages (suspensions/liposomes) when incubated with H441 cell monolayer at air–liquid interface for 4-h (*n* = 4). Phage titer in (**A**) apical chamber, (**B**) cell lysates, and (**C**) percentage phage recovered after 4 h. Data represent four replicates, with treatment groups compared using an unpaired *t*-test (** *p* < 0.01, and **** *p* < 0.0001).

The phage cellular uptake was evaluated in a human lung epithelial cell model (H441 cell monolayer) grown at the air–liquid interface. The TEER value of the cell monolayer was found to be 270 ± 10 Ω.cm^2^, which was consistent with previous literature [28]. Phages (suspension/liposomes) with a titer 2.8 × 10^6^ PFU/mL were incubated with the cell monolayer for four hours. After four-hour incubation, phage liposomes exhibited greater retention (2.59 ± 0.30 × 10^6^ PFU/mL) outside the cells in the apical chamber than the free phages (2.10 ± 0.30 × 10^6^ PFU/mL) (Figure 4A). Consequently, the titer in cell lysates of the phage liposomes (1.42 ± 0.16 × 10^4^ PFU/mL) was significantly (*p* < 0.0001) lower than the free phages (2.91 ± 0.28 × 10^4^ PFU/mL). This suggests that encapsulation of phages in liposomes can reduce the phage cellular uptake by two-fold (Figure 4B). This trend also holds true for the phage titer per µg cell lysate protein being significantly lower (*p* < 0.05) for the phage liposomes (31.68 ± 5.56 PFU) than the free phages (46.26 ± 4.46 PFU). It is interesting to note that the phage recovery (Figure 4C) at the end of the experiment was higher in the phage liposomes (89.39 ± 9.88%) than in the phage suspensions (65.91 ± 7.80%). This indicates that encapsulation in liposomes protects phages from inactivation in this in vitro study environment. No phages were detected in the basal chamber, or the titer may have been below the limit of detection (less than 100 PFU/mL).

### 3.6. Phage Cellular Uptake—Confocal Laser Scanning Microscopy

**Figure 5:**
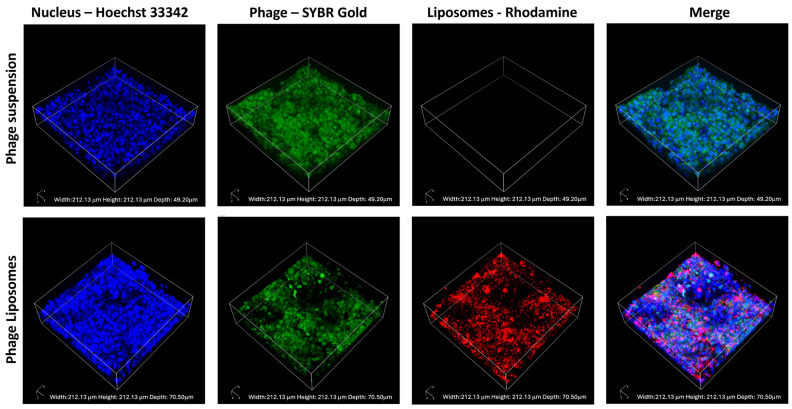
Representative confocal images of H441 cell monolayers after 4-h treatment with phage suspension or liposomes. SYBR Gold-labeled phages in green, Hoechst 33342 stained nuclei in blue, and rhodamine-labeled liposomes in red.

The phage cellular uptake and distribution in the H441 cell monolayer were visualized using confocal laser scanning microscopy. SYBR Gold-labeled phages (green), incubated with H441 cells with Hoechst-stained nuclei (blue), were imaged. Figure 5 shows the confocal images captured after incubating the cell monolayer with the phage suspension (as control) and labeled phage liposomes for four hours. The fluorescence intensity of the phages (green) was higher in the phage suspension than in the liposomes. This indicates that unencapsulated phages (suspension) show greater cellular uptake than the phage liposomes. This also correlates with our observation in the quantitative analysis of phage cellular uptake (Section 3.5).

## 4. Discussion

Liposomal formulations have been explored for pulmonary delivery owing to their potential for controlled delivery and biocompatibility [32,33,34]. The goal of this study was to understand the utility of liposomes for inhaled phage therapy. Phages are highly sensitive and may lose their viability at different checkpoints during inhaled therapy: (i) during manufacturing, (ii) upon aerosolization, and (iii) after deposition on the lung surface [14]. This study focuses on the encapsulation of phages in liposomes for inhalation to target lung infections caused by the extracellular bacteria *Pseudomonas aeruginosa*. We hypothesized that phage encapsulation in liposomes can offer protection to phages from external stressors during manufacturing and delivery. Additionally, we investigated whether liposomal encapsulation could reduce phage cellular uptake in lung epithelial cells, which could result in more viable phages for killing extracellular bacteria.

Liposomes have been studied for phage encapsulation [17,18,19,26,35]. However, challenges such as low encapsulation efficiencies and loss of phage viability during the preparation of liposomes are commonly observed [31]. Previous literature has reported an overestimation of encapsulated phages, as externally bound phages were considered encapsulated phages but physical separation of phages was not performed [35]. Another study formulated phage–liposome nanocomplexes based on the adsorption of phages on the surface of liposomes. However, this adsorption is a dynamic process and may cause reversible changes from bound to unbound phages [19,35]. Our study focused on understanding the influence of hydration titers on encapsulated phage titers and loss of phage viability. We tested two hydration titers (1.98 × 10^7^ and 5.49 × 10^8^ PFU/mL) yielding low-titer (LT) and high-titer (HT) liposomes, respectively. Figure 1B compares the loss in phage titer between the LT and HT liposomes. After the separation of free phages using SEC, no significant loss was observed in LT liposomes, which indicates the presence of fewer phages. Whereas significant loss (** *p* < 0.010) observed in HT liposomes confirms the presence of a greater number of free phages. Exposure to external stress is greatly experienced by free phages compared to encapsulated phages. Consequently, HT liposomes show a greater loss in phage viability than LT liposomes (Figure 1B). Liposomes have a limited loading capacity for encapsulation. Therefore, the increase in phage loading titer does not lead to an increase in liposomal encapsulation. Cinquerrui et al. observed the tendency of phages to aggregate at higher titers (at titers starting from 10^8^ PFU/mL) [35]. This formation of large aggregates can limit the encapsulation inside liposomes due to the limited loading capacity.

The in vitro release of phages from the liposomes was evaluated in the SLF containing the lung surfactant DPPC. The phage viability and release from liposomes in the SLF (at 37 °C) has not been studied previously. Our study demonstrated that the phages remained viable for over 10 h in the SLF, and a slow release of the phages from the liposomes was observed. Slow and controlled release of phages from the liposomes was observed over 8 h. Following this, a steep increase in phage release was observed between 8 and 10 h. A plausible explanation could be the adsorption of DPPC molecules on liposomes, which may facilitate membrane permeabilization and burst release [36]. This effect may be further augmented by the presence of salt ions in SLF, which induce electrostatic interactions with lipid components, leading to membrane destabilization and phage release [37]. Complete release of phages from the liposomes occurred at the end of 10 h. Colom et al. encapsulated three different phages in liposomes for oral therapy and the release of these phages was conducted in the chicken cecal media [17]. The phage-encapsulated liposomes in their study exhibited cationic surface charge, and the phages with long, contractile tails showed slower release. This study highlighted that the phage and lipid interactions can govern its release from liposomes [17]. Hence, the slow release of phages from the liposomes in our study can also be linked to the surface charge of liposomes (anionic), phage morphology, and/or the nature of release media. These observations make it imperative to understand phage–lipid interactions and phage morphology, which can play a key role in the modulation of phage release from liposomes. Further investigation is required to establish a mechanistic understanding of phage release kinetics.

An important aspect in the development of inhaled phage formulations is to determine the delivery of viable phages in the lungs. Nebulization is a common method for converting liquid into fine mist and has been used for liposomal delivery to the lungs [38]. However, the shear stress induced by nebulization can negatively impact phage viability [39]. Our findings show that the liposomes (1.08 ± 0.05 log loss) experienced lower phage titer loss upon nebulization compared to the free phage suspensions (1.55 ± 0.04 log loss) (Table 2). Moreover, our phage liposomes encountered only a ten-fold reduction in phage viability upon nebulization, which is less than the eighteen-fold reduction previously reported for HSPC-cholesterol liposome nanocomplexes [19]. This suggests that a mixture of HSPC and DSPG-Na [40] improves phage stabilization during nebulization compared to HSPC alone. The in vitro deposition profile (Figure 3) showed that the FPF of phage liposomes (37.32 ± 2.94%) was slightly higher than that of the phage suspensions (25.09 ± 8.01%), although the difference was not statistically significant. It is interesting to note that the FPF observed in our study (using a PARI LC Plus nebulizer) was much higher than some previously reported studies using jet nebulizers (Aeroneb Go, AeroEclipse), which showed FPF of less than 12% [14,41,42]. Regardless, it is also important to consider factors such as phage type/morphology, aerosolization conditions, and device, which can affect the respirable phage dose.

Recent studies confirm that phages can be taken up by human epithelial cells [16,26,43]. One of these studies demonstrated extensive accumulation of *Escherichia coli* T4 phages in lung epithelial cells (A549) on cellular uptake, compared to other cell types (endothelial and fibroblast cells) [16]. Such phage cellular uptake can result in fewer phages available extracellularly. The target bacteria in our study, *P. aeruginosa*, is an extracellular pathogen and may exist as aggregates (called biofilms) in chronic infections [44,45]. While a few studies report its intracellular presence [46], the bacteria predominantly remain extracellular. Hence, phage cellular uptake in such extracellular infections may reduce the phage titer available for antibacterial action. Nevertheless, phage uptake may not necessarily be a disadvantage in the case of intracellular bacterial killing. We investigated the cellular phage uptake using an in vitro model consisting of H441 cells (grown at an air–liquid interface) derived from lung adenocarcinoma. Salomon et al. confirmed that H441 cells can form monolayers with appreciable barrier properties, with key transporter expression consistent with human alveolar epithelial cells in primary culture. This study establishes H441 as an alternative to primary alveolar epithelial cells, which experience time-consuming culture conditions and donor variability [28]. In the present study, the liposomal formulation significantly decreased phage cellular uptake and increased free phages in the apical chamber by two-fold compared to the free phage suspensions in the H441 cell monolayer (Figure 4 and Figure 5). This effect can be explained by the presence of PEGylated lipids (DSPE-PEG) on the surface of liposomes. Nunes et al. studied the effect of PEGylation on drug cellular uptake in tumor cells and the results revealed that the PEG chains introduced steric hindrance and reduced cellular interaction, which led to reduced cellular uptake [47].

Another consideration for inhaled phage delivery is the uptake of phages at non-infected (or off-target) sites. Free phages uptaken by the epithelial cells represent a “sink” in phage therapy, as described by Barr et al. [16]. Liposomes demonstrate potential in reducing this off-target cell uptake, thereby improving phage availability in lung airways to combat infections. Furthermore, phages can undergo intracellular degradation on cellular uptake [48]. We observed that phage liposomes showed higher recovery (89.39 ± 9.88%) compared to free phages (65.91 ± 7.80%) after cellular uptake (Figure 4C). This suggests that liposomal encapsulation of phages can reduce uptake and further prevent intracellular degradation compared to free phages. A possible reason for this observation is the slow release of phage liposomes, which can limit uptake and subsequent degradation/inactivation.

Our results demonstrate the potential of liposomes for inhaled phage delivery. Phage encapsulation was achieved using thin film hydration with minimal loss in titer (0.64 ± 0.21 log), resulting in an encapsulated titer of 3.66 × 10^6^ PFU/mL. There is scope for further improvement in the loading capacity of phages in liposomes. This optimization would depend on factors such as lipid type, lipid composition, surface charge, and phage type/morphology. Further exploration of these factors is required to enhance the encapsulated phage titer. The phage cellular uptake studies were performed using an in vitro lung epithelial cell model (H441). This model provides a basic understanding of the uptake phenomenon; however, it does not mimic the complex physiological environment of the lung. Interactions of phages with lung physiological barriers like mucus, macrophages, and epithelial cells in the presence of bacteria are critical determinants of inhaled phage delivery. In vivo studies are necessary to further understand the antibacterial activity of liposomal phage formulations.

## 5. Conclusions

This study focused on understanding the utility of liposomes for inhaled phage therapy. Our results indicate that the phage titer used in liposome preparation governs the encapsulation efficiency and the loss of phage viability during liposome processing. In our study, a lower titer was found to be favorable in achieving higher encapsulation while minimizing loss in phage viability. Our study clearly demonstrated that liposomal formulations offered phage protection during nebulization and in vitro aerosolization. Furthermore, in vitro study results demonstrated that our liposomal formulation enabled prolonged phage release in the SLF and significantly reduced phage cellular uptake in human lung epithelial cells (H441). Our formulations show promising potential for inhaled phage liposomes in treating lung infections caused by *P. aeruginosa*. Further investigations into in vivo efficacy are warranted.

## Figures and Tables

**Table 1:** Particle characterization of phage liposomes (*n* = 3, mean ± SD).

Formulation	Particle Size (nm)	Polydispersity Index (PDI)	Zeta Potential (mV)	Phage Encapsulation Efficiency (%)
HT Liposomes	175.36 ± 3.87	0.036 ± 0.010	−55.47 ± 2.07	25.15 ± 5.67
LT Liposomes	171.13 ± 2.04	0.030 ± 0.004	−50.36 ± 6.07	58.33 ± 6.02

**Table 2:** Effect of nebulization on the phage titer (*n* = 3, mean ± SD).

Sample	Log Phage Titer (PFU/mL)			
	Loaded in Nebulizer	Phages Aerosolized	Phage Recovered	Loss on Nebulization
Phage Suspension	6.18 ± 0.07	6.04 ± 0.01	4.49 ± 0.04	1.55 ± 0.04
Phage Liposomes	5.92 ± 0.04	5.48 ± 0.05	4.40 ± 0.10	1.08 ± 0.05

## Data Availability

The original contributions presented in this study are included in the article material. Further inquiries can be directed to the corresponding authors.
